# Case report: Complex internal mammary to pulmonary artery fistula as a cause of hemoptysis in tuberculosis: Diagnosis and endovascular management using ethylene vinyl alcohol copolymer (Onyx)

**DOI:** 10.4103/0971-3026.76045

**Published:** 2011

**Authors:** Gregory Pierce, Chaitanya Ahuja, Meghna Chadha

**Affiliations:** Department of Vascular and Interventional Radiology, Cleveland Clinic, USA

**Keywords:** Ethylene vinyl alcohol copolymer, hemoptysis, liquid embolic agent

## Abstract

A complex right internal mammary to right pulmonary artery fistula resulting in hemoptysis was successfully treated by embolization with a liquid, nonadhesive, embolic agent - ethylene vinyl alcohol copolymer (Onyx). There were no procedural complications and no recurrence of symptoms has been seen after 2 years of follow-up.

## Introduction

Infectious, chronic inflammatory and neoplastic etiologies of hemoptysis have all been described. Bronchopulmonary and systemic to pulmonary artery fistulas are occasionally encountered as a result of chronic inflammatory states.[1] These systemic, nonbronchial communications with the pulmonary arteries are typically peripheral and usually constitute small but important sources of collateral supply to the pulmonary lesions that provoke hemoptysis.[2] The case that we describe of a right internal mammary to pulmonary artery fistula, involving the lateral segment of the right middle lobe, is unique in its extent, high flow rate and complexity.

## Case Report

A 30-year-old patient of Asian decent was transferred to our institution following recurrent bouts of hemoptysis in which approximately 250-300 ml of blood was expectorated over a span of <3 h. Four years ago, he had been treated for pulmonary tuberculosis. Two earlier episodes, 6 and 4 years earlier, were conservatively managed with antibiotics. He gave a history of necrotizing pneumonia in infancy.

**Figure 1(A, B):**
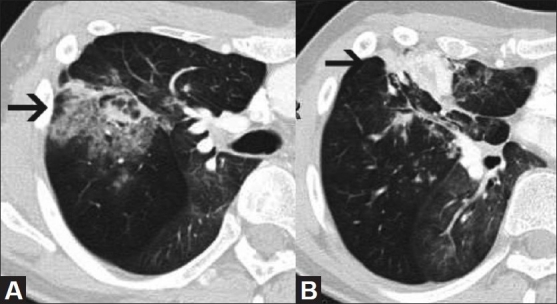
CT scan of the chest at two contiguous levels shows areas of bronchiectasis surrounded by air space opacities (arrow in A) representing hemorrhage within the consolidated right middle lobe, extending to the pleural surface (arrow in B)

**Figure 2:**
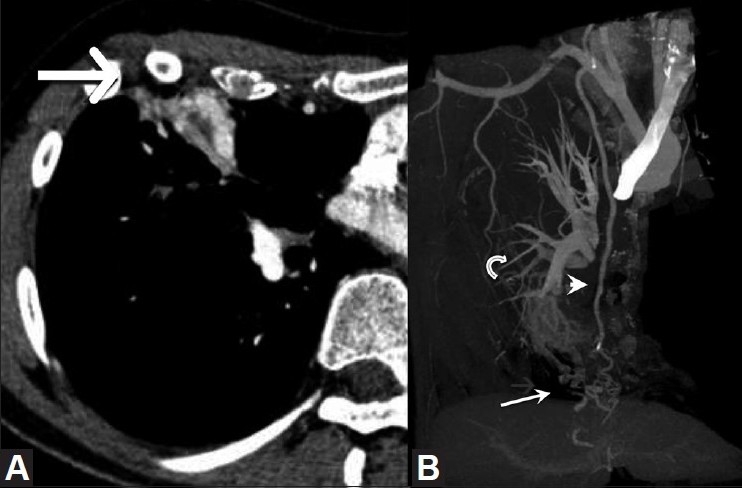
(A, B): Contrast-enhanced CT scan of the chest (A) demonstrates hypervascularity involving the consolidated right middle lobe (arrow). Bone-subtracted, maximum-intensity projection (B) shows a complex right middle lobe vascular malformation (arrow) supplied by the right internal mammary artery (arrowhead) with drainage into the right pulmonary artery (curved arrow)

No fever or elevated white count was present upon arrival to indicate septicemia. A contrast-enhanced CT scan of the chest revealed consolidation and bronchiectasis involving the lateral segment of the right middle lobe and a larger surrounding zone of hazy airspace opacities [Figure 1] probably representing hemorrhage. A hypertrophied right internal mammary artery (IMA) was noted supplying a complex vascular malformation in the right middle lobe [Figure 2] via large pleural and phrenic collaterals draining into the right pulmonary artery. We decided to undertake angiographic evaluation and embolization of this malformation.

**Figure 3:**
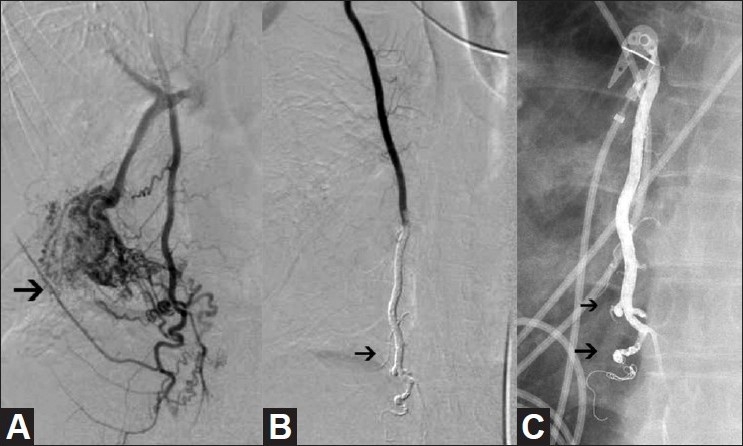
(A-C): Selective right internal mammary artery injection (A) shows the presence of a high-flow right internal mammary artery (IMA) to pulmonary artery malformation (arrow). Selective right IMA arteriogram (B) following coil occlusion of the IMA distal to the lowest contributory branch and Onyx injection shows cessation of flow to the malformation (arrow). Radiograph of the chest (C) the day after embolization shows penetration of Onyx (arrows) into the first-order branches of the IMA

A pigtail oblique thoracic aortogram showed an asymmetrically enlarged right IMA with otherwise normal brachiocephalic arterial and aortic anatomy. More selective injection of the right IMA demonstrated a high-flow plexiform fistulous communication between the right internal mammary and the pulmonary arteries via a plexiform vascular malformation in the right middle lobe [Figure 3A]. Multiple feeders arising from the distal half of the right IMA supplied the malformation. A right bronchial angiogram and contralateral pulmonary and bronchial artery angiograms did not reveal any significant contributors to the malformation. The high-flow rate and large caliber of many of the fistulous communications within the malformation made particulate embolization seem inadvisable. The liquid embolic agent, Onyx, was decided upon as an effective and efficient means of achieving both distal penetration into the malformation and a relatively rapid occlusion of the long segment of the IMA which was supplying the malformation.

An Echelon-14 microcatheter (MTI, Irvine, CA, USA) was advanced coaxially through the existing 5-French diagnostic catheter. The microcatheter was positioned just below the lowest contributory side branch of the IMA and several fibered microcoils, ranging in diameter from 3 mm to 5 mm, were deployed to prevent distal escape of Onyx into the epigastric arteries. After priming of the microcatheter with dimethyl sulfoxide (DMSO) to fill the catheter dead space, Onyx-34 was slowly injected under fluoroscopy over approximately 20 min using a volume sufficient to occlude the distal half of the IMA and the contributory side branches. Due to the viscosity of the selected Onyx, the degree of distal penetration into the malformation was less than what we had anticipated or hoped for. Nevertheless, satisfactory occlusion of the IMA was achieved [Figure 3B, C] without opacification of any arterial feeders from the ipsilateral bronchial artery and contralateral intercostal artery, as demonstrated on the postembolization angiogram. We considered empiric embolization of the right bronchial artery as well, but then decided to await the results of the present embolization before undertaking any further interventions.

On follow-up, the patient has been symptom free for 2½ years.

## Discussion

Bronchopulmonary and systemic pulmonary fistulas have been observed in chronic inflammatory states with both infectious (particularly tuberculosis) and noninfectious etiologies, including postsurgical states following sternotomy. A number of authors have reported the occurrence of fistulous communication in the setting of hemoptysis[1–3] and ischemic coronary steal.[4–6] One case of hemoptysis has also been attributed to a fistula between a left bronchial artery and an internal mammary coronary artery bypass graft.[7]

While bronchopulmonary communications exist in normal lung tissue, where anastomoses occur at the level of the terminal bronchus, nonbronchial systemic arteries must be recruited through the pleura, which explains their appearance in chronic pleuropulmonary diseases. Pulmonary tuberculosis is a well-known culprit. Systemic collaterals may arise from almost any intrathoracic artery as well as any artery passing through the thoracic outlet, including the subclavian, phrenic, axillary, thyrocervical, thoracodorsal and lateral thoracic arteries.[1,2] In our patient, we postulate that the destruction of the pulmonary parenchyma of the right middle lobe secondary to tuberculous disease and parasitization of the systemic arterial supply resulted in a high-flow intrapulmonary shunt. Erosions of high-flow vascular channels within this diseased tissue result in recurring bouts of hemoptysis. While nonbronchial systemic collaterals only occasionally provide the dominant supply to hypervascular pulmonary lesions, the importance of occluding these collaterals has been emphasized by several authors.[1,2] This case is exceptional in the degree of shunting, but this further underscores the importance of these nonbronchial collaterals.

Particulate embolization has been the mainstay of therapy for embolization of both bronchial and nonbronchial collaterals in the setting of hemoptysis. Due to the high-flow nature of the lesion in our patient (more closely resembling an arteriovenous malformation) and the size of the fistulous communications, which we feared would result in particulate passage into the pulmonary arterial tree, we elected to use the liquid embolic agent Onyx.

Onyx is a nonadhesive, radioopaque agent that has FDA approval for use in occluding intracranial vascular malformations. It requires special handling and microcatheters compatible with DMSO. It is commercially available in two viscosities: Onyx-18 and -34. The numbers quantify the viscosity in centipoise. Details of its use and preparation have been described elsewhere.[8] Onyx offers the possibility of deep penetration and near-total occlusion of the nidus, including the potential communications with the bronchial arterial tree.
